# Increased prevalence of peripheral vestibular disorder among patients with Fabry disease

**DOI:** 10.1186/s13023-024-03088-y

**Published:** 2024-03-02

**Authors:** Tzong-Hann Yang, Sudha Xirasagar, Yen-Fu Cheng, Chin-Shyan Chen, Herng-Ching Lin

**Affiliations:** 1 Department of Otorhinolaryngology, Taipei City Hospital, Taipei, Taiwan; 2 Department of Speech, Language and Audiology, National Taipei University of Nursing and Health, Taipei, Taiwan; 3 Department of Exercise and Health Sciences, University of Taipei, Taipei, Taiwan; 4 Research Center of Data Science on Healthcare Industry, Taipei Medical University, Taipei, Taiwan; 5 Department of Health Services Policy and Management, Arnold School of Public Health, University of South Carolina, Columbia, USA; 6 Department of Medical Research, Taipei Veterans General Hospital, Taipei, Taiwan; 7 Department of Otolaryngology-Head and Neck Surgery, Taipei Veterans General Hospital, Taipei, Taiwan; 8 Institute of Brain Science, National Yang Ming Chiao Tung University, Taipei, Taiwan; 9 Department of Otorhinolaryngology, National Yang Ming Chiao Tung University, taipei, Taiwan; 10 Research Center of Data Science on Healthcare Industry, College of Management, Taipei Medical University, Taipei, Taiwan; 11 Department of Economics, National Taipei University, New Taipei City, Taiwan; 12 School of Health Care Administration, College of Management, Taipei Medical University, Taipei, Taiwan; 13 Research Center of Sleep Medicine, Taipei Medical University Hospital, Taipei, Taiwan

**Keywords:** Peripheral vestibular disorder, Prevalence, Vasculopathy, Anderson-Fabry disease

## Abstract

**Background:**

Although peripheral vestibular disorder is a non-fatal complication of Fabry disease, fatalities have been reported in some case reports and case series. To date, no studies have examined the relative risk of peripheral vestibular disorder in patients with Fabry disease compared to the general population without the condition. Due to the high prevalence of Fabry disease in East Asia and the potential shared pathogenic pathways between Fabry disease and vasculopathy, we conducted a study using a nationwide population-based dataset to compare the prevalence of peripheral vestibular disorder between patients with Fabry disease and matched comparison patients.

**Methods:**

Data was sourced from Taiwan’s Longitudinal Health Insurance Database 2010. this study consists of 11,668 sampled patients, 2917 study patients with Fabry disease and 8751 propensity-score-matching comparison patients. We conducted multiple logistic regression analysis to study the association between peripheral vestibular disorder and Fabry disease.

**Results:**

The study identified notable differences in the prevalence of various vestibular disorders between the study and comparison groups. Specifically, there was a 7.2% increased prevalence of peripheral vestibular disorder in the study group (28.3%) compared to the comparison group (20.9%), Meniere’s disease (5.4% vs. 3.7%), benign paroxysmal positional vertigo (5.1% vs. 3.3%), and other/ unspecified peripheral vestibular dizziness (15.6% vs. 11.8%) (all *p* < 0.001). The odds ratios for PVD, MD, BPPV, and other PVD were 1.44 (95% CI = 1.29–1.60), 1.50 (95% CI = 1.23–1.83), 1.59 (95% CI = 1.30–1.95), and 1.40 (95% CI = 1.24–1.58), respectively, among the Fabry disease group relative to the comparison group after adjusting for age, monthly income, geographic location, urbanization level, hyperlipidemia, diabetes, coronary heart disease, and hypertension.

**Conclusion:**

This study found that patients with Fabry disease had increased prevalence of peripheral vestibular disorder.

## Background

Fabry disease, also known as Anderson-Fabry disease, is a rare and progressive X-linked glycosphingolipidosis occurring exclusively among males, with documented prevalence rates ranging from 1:8,454 males to 1:117,000 worldwide [1]. These estimates may be on the lower side because screening in newborns show rates ranging from one in 8800 male newborns to 1:600 to 1:1250 reported in Taiwan [2]. Fabry disease is caused by mutations in the GLA gene at Xq21.3-q22, which encodes the alpha-galactosidase, an enzyme responsible for breaking down toxic alpha-galactose groups formed from glycoproteins and glycolipids. Deficiency or absence of the enzyme results in the systemic and progressive lysosomal accumulation of toxic metabolites such as globotriaosylceramide (Gb3) and related glycosphingolipids such as globotriaosylsphingosine (LysoGb3) in cells of the blood vessels, skin, kidney, heart, and nerve cells, potentially causing cochleovestibular symptoms which could involve hearing loss and vertigo.

Vertigo is a common condition causing approximately 4% of all emergency department visits in the United States. Most cases of vertigo, about 80%, are caused by local pathology of the ear and vestibular organs [3]. Peripheral vestibular disorders (PVD) affect about 1.2–6.5% of the total population causing considerable economic loss due to missed workdays or early retirement [4], as well as causing personal life disruptions, anxiety, social phobias, traffic accidents, increased risk of injuries [5], and a high risk of reoccurrence of vertigo [6]. Vertigo can be attributed to inner ear pathology, which disrupts the coordinated sensory inputs from the visual, vestibular, and proprioceptive systems responsible for balance and spatial orientation. The most common causes of PVD are benign paroxysmal positional vertigo (BPPV), vestibular neuritis (VN), and Meniere disease (MD) [4]. While the exact origins of PVD remain a mystery, there are speculations that ischemia could play a role [7–9]. PVDs are attracting more attention because they cause significant morbidity in Fabry disease. Although PVD is a non-fatal complication of Fabry disease, fatalities have been reported in some case reports and case series [10]. Thus far, the relative risk of the PVD in Fabry disease patients compared to the general population without Fabry disease has not been studied. Given the high prevalence of Fabry disease in East Asia reported in the recent literature [2], and shared pathogenic pathways between Fabry disease and vasculopathy, we used a nationwide population-based dataset to compare the prevalence of PVD among patients with Fabry disease and matched comparison patients with similar vascular risk factors such as hypertension, coronary heart disease and hyperlipidemia.

## Methods

### Database

The cross-sectional study sample for this retrospective observational study was retrieved from Taiwan’s Longitudinal Health Insurance Database 2010 (LHID2010). Taiwan has implemented a universal coverage, single-payer NHI program for all Taiwan citizens since 1995. The LHID2010 contains the medical claims and registration files of 2,000,000 NHI beneficiaries, randomly sampled from the year 2010 Registry of NHI Beneficiaries. The National Health Research Institutes of Taiwan reports that the LHID2010 is a representative sample of the Taiwan NHI beneficiary population on gender, age, and average payroll-related insurance deductions. Many researchers from Taiwan have used deidentified data from the LHID2010 for epidemiological and clinical care research.

The study was approved by the institutional review board of Taipei Medical University (TMU-JIRB N202203211) and is compliant with the Declaration of Helsinki. Because we used deidentified administrative data, informed consent was waived.

### Identification of cases and controls

We identified a study group (cases of Fabry disease) and a comparison group. We identified 2917 patients aged ≥ 20 years old who had received a first-time diagnosis of Fabry disease (ICD-9-CM 272.7 or ICD10 E75.21) during an ambulatory care visit between January 2013 and December 2018. We identified comparison patients from the remaining LHID2010 enrollees from the Registry of beneficiaries, those who did not have a diagnosis of Fabry disease prior to the study period, propensity score matched with Fabry disease patients and selected to achieve a ratio of 3 comparison patients to each Fabry disease case. For propensity score matching we used the available sociodemographic characteristics (age, monthly income, geographic location and urbanization level of the patient’s residence), and the widely prevalent medical conditions, hyperlipidemia, diabetes, coronary heart disease, and hypertension. We entered these variables into a multivariable logistic regression model as predictors of Fabry disease to calculate the propensity score for Fabry disease for each enrollee. We then grouped all patients into quintiles according to propensity scores, and the method of nearest neighbor within calipers of 0.2 was used to match comparison patients. Ultimately, this study consists of 11,668 sampled patients, 2917 study patients and 8751 comparison patients.

### Outcome of interest

The outcome of interest was the prevalence of PVD, defined as proportions of Fabry disease and comparison group showing a diagnosis of PVD in any outpatient claim between 2013 and 2018, the date of claim either preceding or succeeding the date of first Fabry disease diagnosis. We identified PVD cases using ICD diagnosis codes: Meniere’s Disease (ICD-9-CM 386.0 or ICD-10-CM H81.0), BPPV (ICD-9 386.11 or ICD-10 code H81.10), VN (ICD-9 386.12 or ICD-10 H81.2), other/ unspecified peripheral vestibular dizziness (other PVD) (ICD-9 386.10, 386.19, 386.9 or ICD-10 H81.31, H81.39, H81.9, H83.9).

### Statistical analysis

The SAS system (SAS for Windows, V, 8.2, SAS Institute, Cary, NC) was used for statistical analyses, which consisted of chi-square and t tests to examine differences in demographic characteristics and medical comorbidities between the study patients and comparison patients. We conducted multiple logistic regression analysis to study the association between PVD and Fabry disease adjusted for age, monthly income, geographic location, urbanization level, hyperlipidemia, diabetes, coronary heart disease, and hypertension. We used two-tailed *p*≤0.05 to determine statistical significance.

## Results

The mean age of the sampled patients (total 11,668) was 59.8 years (± 14.8 years). Table 1 presents the demographic characteristics and medical co-morbidities of the study group with Fabry disease and (propensity score-matched) comparison group, showing that the two groups were similar on all the matching variables (age, sex, monthly income, geographic location, urbanization level, hyperlipidemia, diabetes, coronary heart disease, and hypertension.

**Table 1:** Demographic characteristics and medical co-morbidities of sampled patients classified by Fabry disease status in Taiwan (*n* = 11,668)

Variable	Patients with Fabry disease (*n* = 2917)		Comparison Patients (*n* = 8751)		*p* value
	Total no.	%	Total no.	%	
Age, mean (SD)	59.8 (14.8)		59.8 (14.7)		0.971
Males*	1305	44.7	3915	44.7	> 0.999
Monthly Income					0.628
<NT$1 ~ 15,841	628	21.5	1822	20.8	
NT$15,841 ~ 25,000	1061	36.4	3255	37.2	
≥NT$25,001	1228	42.1	3674	42.0	
Geographic region					0.561
Northern	1434	49.2	4302	49.2	
Central	411	14.1	1249	14.3	
Southern	990	33.9	2995	34.2	
Eastern	82	2.8	205	2.3	
Urbanization level					0.971
1 (most urbanized)	752	25.8	2267	25.9	
2	955	32.7	2915	33.3	
3	539	18.5	1583	18.1	
4	310	10.6	911	10.4	
5 (least urbanized)	361	12.4	1075	12.3	
Diabetes	1240	42.5	3720	42.5	> 0.999
Hypertension	1772	60.8	5316	60.8	> 0.999
Coronary heart disease	809	27.7	2427	27.6	> 0.999
Hyperlipidemia	2194	75.2	6582	75.2	> 0.999

*No females were identified with Fabry disease in the database, consistent with its origin in an X−linked chromosomal disorder

Table 2 shows statistically significant differences in the prevalence of PVD between the two groups (28.3% vs. 20.9% among the study group and comparison group respectively), MD (5.4% vs. 3.7%), BPPV (5.1% vs. 3.3%), and other PVD (15.6% vs. 11.8%), all *p* < 0.001. There was no difference in the prevalence of VN (2.2% vs. 2.1%, *p =* 0.112) between the two groups. Table 2 also presents the crude and covariate-adjusted odds ratios for PVD among the Fabry disease group relative to the comparison group. The adjusted odds ratios (OR) for PVD, MD, BPPV, and other PVD were 1.44 (95% CI = 1.29–1.60), 1.50 (95% CI = 1.23–1.83), 1.59 (95% CI = 1.30–1.95), and 1.40 (95% CI = 1.24–1.58), respectively, after adjusting for the above covariates. The adjusted OR for VN was not significant (1.05, 95% CI = 0.79 ~ 1.40).

**Table 2:** Crude odds ratios and covariate-adjusted odds of PVD among patients with Fabry disease relative to comparison patients

Variable	Patients with Fabry disease	Comparison Patients
	Total	
	(*n* = 2917)	(*n* = 8751)
Presence of PVD	825 (28.3%)	1829 (20.9%)
Crude OR (95% CI)	1.405 (1.266 ~ 1.560)	1.000
Adjusted OR^a^ (95% CI)	1.439 (1.292 ~ 1.603)	1.000
Presence of Meniere’s disease	157 (5.4%)	324 (3.7%)
Crude OR (95% CI)	1.480 (1.217 ~ 1.799)	1.000
Adjusted OR^a^ (95% CI)	1.500 (1.229 ~ 1.829)	1.000
Presence of benign paroxysmal positional vertigo	148 (5.1%)	286 (3.3%)
Crude OR (95% CI)	1.582 (1.291 ~ 1.938)	1.000
Adjusted OR^a^ (95% CI)	1.590 (1.295 ~ 1.952)	1.000
Presence of vestibular neuritis	64 (2.2%)	183 (2.1%)
Crude OR (95% CI)	1.050 (0.788 ~ 1.400)	1.000
Adjusted OR^a^ (95% CI)	1.051 (0.787 ~ 1.403)	1.000
Presence of other/ unspecified peripheral vestibular dizziness	456 (15.6%)	1036 (11.8%)
Crude OR (95% CI)	1.380 (1.225 ~ 1.555)	1.000
Adjusted OR^a^ (95% CI)	1.402 (1.241 ~ 1.584)	1.000

*Notes: CI = confidence interval; OR = odds ratio;* ^a^ *Adjusted for age, monthly income, geographic location, urbanization level, hyperlipidemia, diabetes, coronary heart disease, and hypertension*

## Discussion

To the best of our knowledge, this is the first large-scale, population-based epidemiological study comparing the prevalence of PVD among patients with and without Fabry disease. Our results show that Fabry disease is significantly associated with peripheral vestibular diseases (OR = 1.439), the difference being reflected in MD, BPPV, and other PVD. We found that the odds of MD are 50% higher among persons with Fabry disease than among comparison patients, 59% higher for BPPV, and 40% higher for other PVDs excluding vestibular neuritis.

Our study also showed that peripheral vertigo occurred in 28.3% of patients with Fabry disease. Prior studies have documented high rates of vertigo or dizziness among persons with Fabry disease, ranging between 10.7% and 54% [11–14], without, however, distinguishing true vertigo from non-specific dizziness, and between peripheral causes of vertigo from central lesion-caused vertigo. Patients with Fabry disease may be more susceptible to cardiovascular or cerebrovascular disease [15], which may also cause dizziness and fainting, mimicking vertigo from vestibular diseases. Nevertheless, patients with Fabry disease have manifested high susceptibility to impaired vestibular tests such as head impulse test (HIT) [14, 16, 17], electro-/video-nystagmography (ENG/VNG) [14, 18], caloric testing [16, 18, 19], or vestibular evoked myogenic potentials (VEMP) [16, 19]. Progressive deterioration of vestibular function is also reported, affecting 80% of men and 77% of women when assessed with head impulse testing [16]. Moreover, the vestibular function loss may lead to ambulatory instability or a fall because of poor postural control [20].

The anatomic site of vestibulo-cochlear damage in Fabry disease is not known. Although otological symptoms are quite common in patients with Fabry disease [2], there is only one documented report on human temporal bone pathology in the world [21]. In their first patient, the utricles and vestibules revealed no morphological abnormalities, but in the non-ampullated end of the superior semicircular canal, new bone filled the perilymphatic space in the first patient. In addition, vestibular ganglia of the right ear exhibited ballooning of the ganglion cells. In the second patient, a mild hemorrhage was found within the saccule of the left ear. Ganglion cells exhibited a ballooning appearance with granulating nuclei in the vestibular ganglia. Some studies did not found involvement of eighth cranial nerve, and postulated that cochleovestibular ischemia caused by lipid accumulations in the endothelial cells may explain the vertigo [14, 19].

### Fabry disease is associated with Meniere’s Disease

Our study finds that patients with Fabry disease have a higher risk of Meniere’s disease (MD), with a prevalence of 5.4%, which is lower than the rate reported by previous studies (9.1- 32%) [12, 22]. MD is a chronic condition of the inner ear characterized by recurring vertigo, fluctuating hearing loss, tinnitus, and a sensation of fullness in the ear [23]. The underlying cause of MD is not well understood, but it is believed to be a multifactorial disorder involving several factors, including genetic abnormalities, ionic imbalance, viral infections, autonomic imbalance, dietary factors, autoimmune reactions, and vascular irregularities [9].

Ischemia has been suggested as a potential cause of MD symptoms [24]. The similarities observed in symptom presentation and vascular events during an attack between Meniere’s disease, migraines, and allergies [25] support this hypothesis. Some researchers have proposed that vasospasm of small vessels in the inner ear may be responsible for migraine-associated vestibular symptoms. Migraine occurs more commonly in patients with MD than in the general population, leading to the postulation of a common vascular pathophysiology for the two disorders [26, 27]. Additionally, epidemiologic studies suggest a possible association between MD and high cardiovascular risk [28]. Vascular involvement of Fabry disease could lead to ischemia of the labyrinthine organ and, consequently, induce MD or mimic its symptoms.

### Fabry disease is associated with benign paroxysmal positional vertigo

Our study finds that patients with Fabry disease have a higher risk of BPPV, with a prevalence of 5.1%, somewhat lower than the reported rates in prior studies (7.1-10%) [18, 29]. The mechanism of BPPV causation is thought to be dislodged calcium debris [30], which can leave the utricle and float freely in the semicircular canals or attach to the cupula, making the labyrinth sensitive to gravitational forces. The debris likely represents loose otoconia (calcium carbonate crystals) originating from the utricular sac [30]. However, the pathogenesis of calcium debris and its dislodgement is not fully understood. Several conditions, such as sudden deafness [31, 32], migraine [33], Meniere disease [32, 33], and giant cell arteritis [34], have also been associated with BPPV and have been linked to inner ear ischemia. One population-based survey suggested that BPPV was independently associated with age, migraine, hypertension, hyperlipidemia, and stroke, which may suggest a vascular mechanism for the development of BPPV [35]. Based on these findings, it is possible that the vascular involvement seen in Fabry disease may contribute to the development of dislodged calcium debris, causing BPPV as observed in our study. Further research is needed to fully understand the underlying mechanisms of this association.

### Fabry disease is not associated with vestibular neuritis

Our study found no evidence that patients with Fabry disease may be at a higher risk of vestibular neuritis (VN), consistent with most studies, except for one case series reporting vestibular neuritis in 2.9% of patients [11]. Vestibular neuritis is a type of acute peripheral vestibular syndrome that is characterized by sudden onset of severe vertigo, nausea, vomiting, and gait instability, with rapid and spontaneous recovery. It is defined by an acute, unilateral loss of peripheral vestibular function without evidence of acute central neurological or audiological symptoms or signs. Vestibular neuritis is generally believed to be caused by a viral or post-viral inflammatory disorder affecting the vestibular portion of the eighth cranial nerve [36], and some pathologic data support this mechanism [37, 38]. A history of preceding viral illness is often reported by patients, and magnetic resonance imaging studies have revealed patterns of enhancement consistent with an inflammatory process in patients with VN [39]. While some studies have suggested a possible vascular etiology for VN [8, 40], our study found no evidence to suggest that Fabry disease-related vascular involvement contributes to VN.

This study has several strengths. Firstly, it utilizes data on a large-scale, population-wide sample of patients from a nationwide dataset. Therefore the findings cover the entire population and can be generalized to a broader context. Moreover, the Taiwanese healthcare system is both easily accessible and affordable for all residents due to very low copayments, reducing the risk of selection bias based on socioeconomic status or residential location, and allows for a more socio-economically diverse sample of patients. Another strength is the ability to identify patients with Fabry disease and peripheral vestibular disease from all sources, as the national health insurance (NHI) claims data covers all healthcare utilization episodes of all Taiwanese residents. Patients with both serious and minor medical conditions, such as Fabry disease and vertigo, can seek medical attention without delay and get diagnosed. Additionally, the use of NHI claims data circumvents recall bias, a weakness of self-reported survey data. Finally, cross-sectional study design is a strength, with comparison patients selected by propensity score matching. These factors strengthen the validity of the findings and supports inference of a real association between Fabry syndrome and vertigo with minimal selection bias and misclassification bias.

Some limitations should be noted. Claims data are subject to coding errors. Recorded peripheral vestibular diseases identified through International Classification of Diseases (ICD) codes may not be as accurate as diagnoses recorded in electronic medical records (EMRs) based on standardized clinical examinations. Absence of valid and reliable diagnosis may cause misclassification bias, such as central vertigo misdiagnosed as peripheral vertigo in some cases. However, any misclassification bias is likely to be random and may not significantly impact the validity of findings. Another limitation is the lack of data on confounding variables, such as family history, environmental factors (for example: stress, lifestyle, diet, minor head trauma), genetic factors, race, and laboratory data related to Fabry disease and vertigo. While our study is population-based and includes a large and socio-economically diverse sample, these variables could impact the relationship between Fabry disease and vertigo in other ethnicities/national economic conditions, potentially limiting generalizability of our findings to other regions or countries.

## Conclusion

Our study revealed a heightened occurrence of peripheral vestibular disorders in patients with Fabry disease when compared to their counterparts. However, vestibular neuritis was an exception. It is imperative for physicians to be vigilant about potential otoneurologic symptoms in these patients. A meticulous differential diagnosis is essential to pinpoint the root cause of the peripheral vestibular disorder in this demographic.

## Data Availability

Data from the National Health Insurance Research Database, now managed by the Health and Welfare Data Science Center (HWDC), can be obtained by interested researchers through a formal application process addressed to the HWDC, Department of Statistics, Ministry of Health and Welfare, Taiwan (https://dep.mohw.gov.tw/DOS/lp-2506-113.html. 02/01/2022).

## Abbreviations

PVD: Peripheral vestibular disorders
BPPV: Benign paroxysmal positional vertigo
VN: Vestibular neuritis
MD: Meniere disease
LHID2010: Longitudinal Health Insurance Database 2010
